# Data on somatic mutations obtained by whole exome sequencing of FFPE tissue samples from Russian patients with prostate cancer

**DOI:** 10.1016/j.dib.2019.104022

**Published:** 2019-05-24

**Authors:** A.S. Nikitina, E.I. Sharova, S.A. Danilenko, O.V. Selezneva, L.O. Skorodumova, A.V. Kanygina, K.A. Babalyan, A.O. Vasiliev, A.V. Govorov, E.A. Prilepskaya, D.Y. Pushkar, E.S. Kostryukova, E.V. Generozov

**Affiliations:** a Federal Research and Clinical Center of Physical-Chemical Medicine of Federal Medical Biological Agency, Moscow, Russia; b Moscow Institute of Physics and Technology, Dolgoprudnyi, Russia; c Department of Urology, Moscow State Medical Stomatological University, Moscow, Russia

**Keywords:** Prostate cancer, Somatic variants, Whole exome

## Abstract

Prostate cancer (PCa) is the most frequently diagnosed among men malignant disease that remains poorly characterized at the molecular level. Advanced PCa is not curable, and the current treatment methods can only increase the life expectancy by several months. Identification of the genetic aberrations in tumor cells provides clues to understanding the mechanisms of PCa pathogenesis and the basis for developing new therapeutic approaches. Here we present data on somatic mutations, namely single nucleotide variations (SNVs), small insertions and deletions, detected in prostate tumor tissue obtained from Russian patients with PCa. Moreover, we provide a raw dataset on the whole exome and targeted DNA sequencing of tumor and non-tumor prostate tissue obtained from Russian patients with PCa and benign prostatic hyperplasia (BPH). This data is available at NCBI Sequence Read Archive under Accession No. PRJNA506922.

**Table:** Specifications Table

Subject area	Biology
More specific subject area	Prostate cancer research
Type of data	Text (FASTQ sequence files, VCF file), tables, figures
How data was acquired	High-throughput sequencing using Ion Proton System (Thermo Fisher Scientific)
Data format	Raw and analyzed
Experimental factors	Prostate tissue samples were obtained after radical prostatectomy or transurethral resection of prostate (TURP) from patients with PCa and BPH respectively. The postoperative material was fixed in formalin and embedded in paraffin (FFPE).
Experimental features	DNA was isolated from FFPE tissue using AllPrep DNA/RNA FFPE and GeneRead DNA FFPE kits (Qiagen). Whole exome libraries were constructed with Ion AmpliSeq Exome RDY Kit (Thermo Fisher Scientific). Targeted DNA enrichment was performed by GeneRead DNAseq Targeted Human Prostate Cancer Panel.
Data source location	Moscow, Russia
Data accessibility	Raw data was deposited at NCBI SRA database under accession No. PRJNA506922 https://www.ncbi.nlm.nih.gov/bioproject/PRJNA506922

**Table:** 

**Value of the data** • Detection of somatic mutations by whole exome sequencing is the widely recognized method used to identify genetic abnormalities in tumors for various types of cancer [1], [2], [3]. The data on somatic mutations presented here can serve as the basis for studying the pathogenesis of the disease and the search for new therapeutic targets. • The dataset on targeted DNA sequencing also presented here could be valuable for reliable validation of identified somatic mutations due to the much higher coverage compared to whole exome sequencing. • Data on samples from patients with BPH may be used within a control group for validation of the detected genetic variants to identify mutations specific to malignant prostate tissue. • The same tissue samples were previously subjected to transcriptome profiling by RNA sequencing [4]. Moreover, urine and plasma from these patients was also used for total RNA and targeted DNA sequencing [5]. Thus, this dataset can be valuable for an integrated analysis of DNA and RNA sequencing data obtained from PCa and BPH patients' multiple tissues. • The dataset can be readily incorporated into the study involving other sample cohorts and implementing any computational algorithms of choice since the data is available in raw format and the metadata includes comprehensive clinical patient information (serum PSA level, Gleason grade, TNM clinical and pathological stage, extraprostatic extension, seminal vesicles and perineural invasion, surgical margins status).

## 1 Data

Matched tumor and non-tumor FFPE prostate tissue samples were obtained from 26 patients with PCa and 8 patients with BPH via radical prostatectomy or TURP, respectively. DNA extracted from these samples was used to construct 61 whole exome and 25 targeted DNA libraries that were sequenced using Ion Proton platform. The corresponding raw sequencing data (reads in FASTQ format) was deposited at NCBI SRA database under project accession No. PRJNA506922.

The data on whole exome sequencing of samples from PCa patients (50 matched samples from 25 patients) was analyzed to detect somatic mutations in prostate cancer tissue. Reads were mapped to the GRCh37 assembly of the human genome. Paired variant calling performed for matched samples allowed to filter germline mutations and detect somatic variants in tumor tissue. The information on identified somatic alterations is presented in VCF format in Supplementary File 1. A total of 1696 somatic mutations in all 25 tumor samples were detected, including 1686 (99.4%) SNVs, 8 (0.472%) insertions and 3 (0.118%) deletions. The summary of detected somatic variants is shown in Table 1.

**Table 1:** Types of somatic variants detected in FFPE prostate cancer tissue samples.

	Median	Min	Max
Total variants	29	9	301
Mutated genes	29	9	296
Genes potentially affected (including intergenic effects)	44	12	437
SNV	29	9	291
Insertions	0	0	20
Deletions	0	0	38
Missense	13	3	123
Synonymous	5	1	42
Stop gained	1	0	7
Start lost	0	0	2

**Fig. 1:**
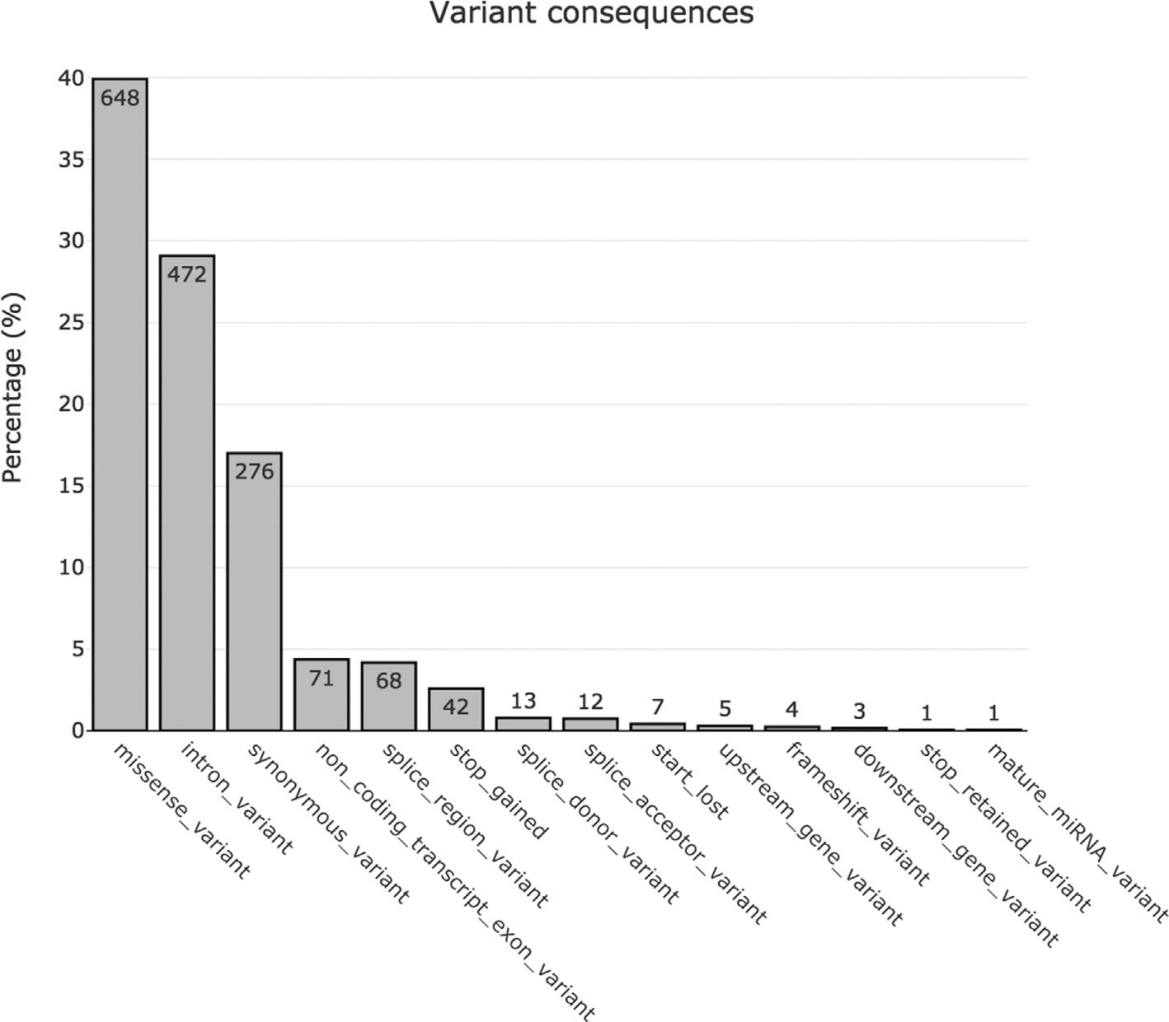
Summary of variant consequences predicted by VEP.

Moreover, variant annotation was performed using Variant Effect Predictor (VEP) which identifies genes and transcripts affected by genetic alterations and predicts their consequences on protein sequences (Fig. 1).

**Fig. 2:**
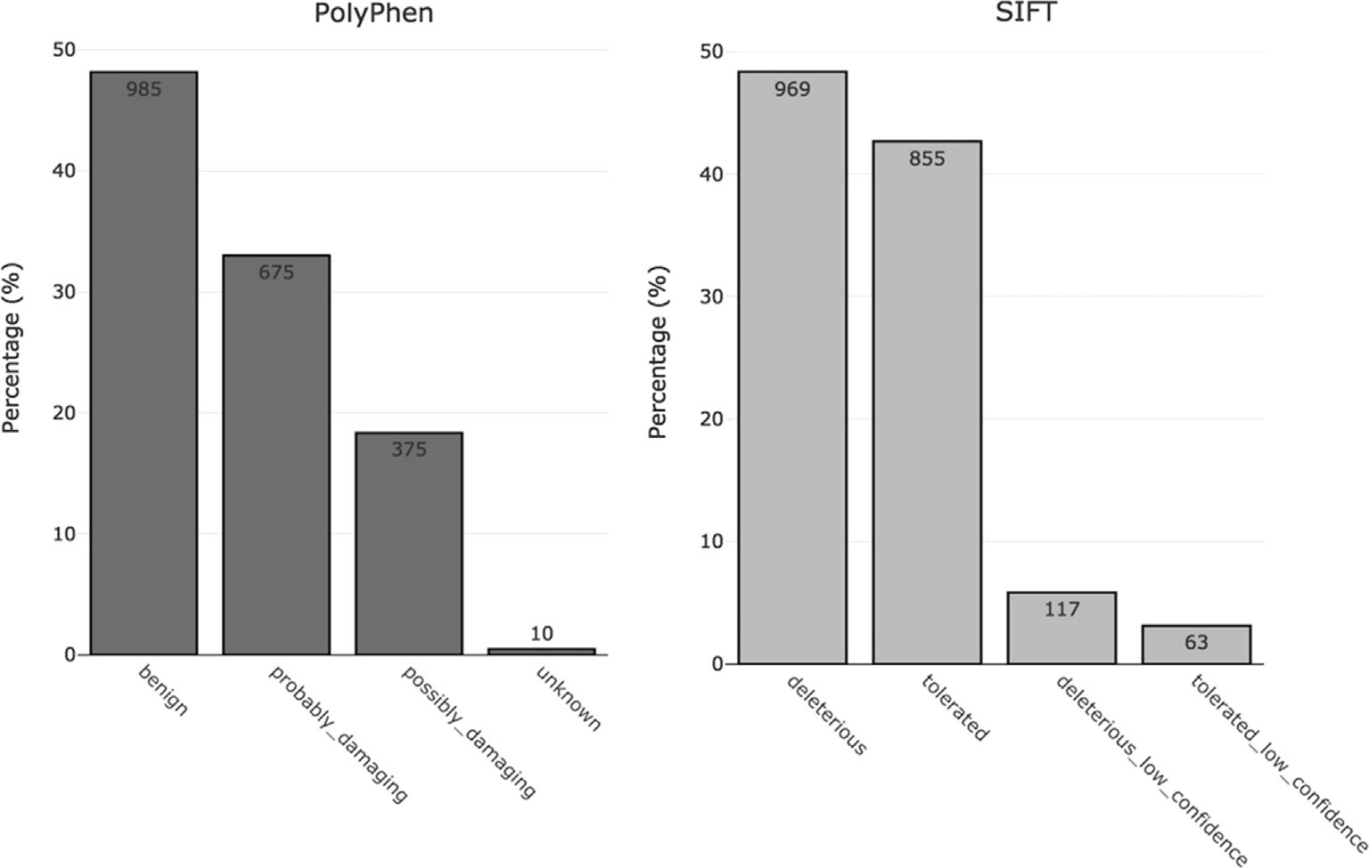
Summary of PolyPhen and SIFT predictions.

In addition, SIFT and PolyPhen algorithms were implemented to predict the effect of amino acid substitution caused by a variant on the structure and function of a protein (Fig. 2). The VEP annotation of each variant is included in the Supplementary File 1. The data used to draw bar charts is presented in Supplementary Table 1.

## 2 Experimental design, materials, and methods

### 2.1 Sample collection and DNA extraction

All patients had signed an informed consent form. Tissue samples were obtained from 26 patients with PCa and 8 patients with BPH from City Clinical Hospital No. 50 via radical prostatectomy or TURP, respectively. All patients had not received specific therapy prior to sample collection. Clinical patient data including serum PSA level, Gleason grade, TNM clinical and pathological stage, extraprostatic extension, seminal vesicles and perineural invasion, surgical margins status is provided in Supplementary Table 2. The postoperative material was fixed in formalin and embedded in paraffin, the corresponding thin sections of the FFPE tissue samples were examined by the pathologist determined areas of tumor and non-tumor adjacent tissue. DNA was extracted from these marked regions using AllPrep DNA/RNA FFPE and GeneRead DNA FFPE kits (Qiagen). Table 2 provides information about samples, DNA extraction kits used and corresponding libraries. For each patient maximum of two DNA samples were obtained: from tumor and non-tumor adjacent tissue. Either exome or targeted panel library or both were constructed from each DNA sample. Every library name corresponds to a single library and to a single FASTQ record in NCBI SRA database.

**Table 2:** Sample information.

Patient ID	Age	Diagnosis	Libraries from pathological tissue	Libraries from non-pathological tissue	DNA extraction kit
A50_001	75	BPH	PC1015D_exome PC1015D_panel	PC1016D_exome PC1016D_panel	AllPrep DNA/RNA FFPE Kit
A50_002	67	BPH	PC1009D_exome PC1009D_panel	PC1010D_exome PC1010D_panel	AllPrep DNA/RNA FFPE Kit
A50_003	58	BPH	PC1007D_exome PC1007D_panel	PC1008D_exome PC1008D_panel	AllPrep DNA/RNA FFPE Kit
A50_004	68	BPH	PC1013D_exome	–	AllPrep DNA/RNA FFPE Kit
A50_006	64	prostate cancer	PC1019D_exome	PC1020D_exome	AllPrep DNA/RNA FFPE Kit
A50_010	69	BPH	PC1039D_exome	–	AllPrep DNA/RNA FFPE Kit
A50_011	63	BPH	PC1083D_exome	–	GeneRead DNA FFPE
A50_012	84	BPH	PC1085D_exome	–	GeneRead DNA FFPE
A50_017	50	BPH	PC1087D_exome	–	GeneRead DNA FFPE
P50_001	60	prostate cancer	PC1003D_exome	PC1004D_exome	AllPrep DNA/RNA FFPE Kit
P50_002	55	prostate cancer	PC1001D_exome	PC1002D_exome PC1002D_panel	AllPrep DNA/RNA FFPE Kit
P50_003	61	prostate cancer	PC1005D_exome PC1005D_panel	PC1006D_exome PC1006D_panel	AllPrep DNA/RNA FFPE Kit
P50_004	55	prostate cancer	PC1031D_exome PC1031D_panel	PC1032D_exome PC1032D_panel	AllPrep DNA/RNA FFPE Kit
P50_005	61	prostate cancer	PC1011D_exome	PC1012D_exome	AllPrep DNA/RNA FFPE Kit
P50_006	67	prostate cancer	PC1041D_exome PC1041D_panel	PC1042D_exome PC1042D_panel	AllPrep DNA/RNA FFPE Kit
P50_008	69	prostate cancer	PC1033D_exome PC1033D_panel	PC1034D_exome PC1034D_panel	AllPrep DNA/RNA FFPE Kit
P50_009	57	prostate cancer	PC1027D_exome PC1027D_panel	PC1028D_exome PC1028D_panel	AllPrep DNA/RNA FFPE Kit
P50_010	69	prostate cancer	PC1023D_exome PC1023D_panel	PC1024D_exome PC1024D_panel	AllPrep DNA/RNA FFPE Kit
P50_011	67	prostate cancer	PC1025D_exome	PC1026D_exome	AllPrep DNA/RNA FFPE Kit
P50_012	68	prostate cancer	PC1021D_exome	PC1022D_exome	AllPrep DNA/RNA FFPE Kit
P50_013	56	prostate cancer	PC1017D_exome PC1017D_panel	PC1018D_exome PC1018D_panel	AllPrep DNA/RNA FFPE Kit
P50_015	48	prostate cancer	PC1029D_panel	PC1030D_panel	AllPrep DNA/RNA FFPE Kit
P50_016	67	prostate cancer	PC1035D_exome	PC1036D_exome	AllPrep DNA/RNA FFPE Kit
P50_018	69	prostate cancer	PC1055D_exome	PC1056D_exome	GeneRead DNA FFPE
P50_019	73	prostate cancer	PC1057D_exome	PC1058D_exome	GeneRead DNA FFPE
P50_020	50	prostate cancer	PC1047D_exome PC1047D_panel	PC1048D_exome PC10478_panel	AllPrep DNA/RNA FFPE Kit
P50_022	67	prostate cancer	PC1037D_exome	PC1038D_exome	AllPrep DNA/RNA FFPE Kit
P50_023	67	prostate cancer	PC1059D_exome	PC1060D_exome	GeneRead DNA FFPE
P50_024	65	prostate cancer	PC1061D_exome	PC1062D_exome	GeneRead DNA FFPE
P50_027	61	prostate cancer	PC1071D_exome	PC1072D_exome	GeneRead DNA FFPE
P50_028	58	prostate cancer	PC1063D_exome	PC1064D_exome	GeneRead DNA FFPE
P50_031	65	prostate cancer	PC1065D_exome	PC1066D_exome	GeneRead DNA FFPE
P50_033	60	prostate cancer	PC1073D_exome	PC1074D_exome	GeneRead DNA FFPE
P50_036	40	prostate cancer	PC1075D_exome	PC1076D_exome	GeneRead DNA FFPE

### 2.2 Whole exome library preparation

Amplification of exonic regions was performed using Ion AmpliSeq Exome RDY Kit (Thermo Fisher Scientific). Considering the quality of FFPE-derived DNA the number of cycles in this amplification step was raised to 13–15 instead of 10 recommended by the manufacturer. Further steps of library preparation were carried out in accordance with the manufacturer's instructions.

### 2.3 Targeted DNA library preparation

GeneRead DNAseq Targeted Human Prostate Cancer Panel (Qiagen) was used for targeted enrichment of the extracted DNA. This amplification procedure was also modified as for exome libraries to account for DNA quality extracted from FFPE tissue samples. Number of PCR cycles was raised to 20–22 instead of 18 recommended for standard DNA samples. Subsequent library construction was performed using GeneRead Library Prep workflow (Qiagen) following the manufacturer's recommendations.

### 2.4 High-throughput sequencing

Quality of the constructed libraries was assessed by 2100 Bioanalyzer (Agilent Genomics) using Agilent High Sensitivity DNA Kit (Agilent Genomics). High-throughput sequencing was performed on Ion Proton platform using ION PI HI-Q Sequencing 200 Kit and Ion PI Chip Kit v2 (Thermo Fisher Scientific) following the recommendations of the manufacturer. Base calling was performed by Torrent Suite 5.0, fastqCreator v3.4.56313.

### 2.5 Detection and annotation of somatic variants

Reads were mapped to the human genome (GRCh37 assembly) with *bwa mem* tool from BWA package with the following non-default parameters: -c 250 -M [6]. Paired somatic calling was performed using 4 variant callers: MuTect (v. 1.1.7) [7], freebayes (v. 1.0.2) [8], VarDict (v. 2016.02.19) [9] and VarScan (v. 2.4.1) [10] which were run via bcbio-nextgen (v. 0.9.7) somatic variant calling pipeline [11] with minimal allele fraction equal to 0.1. The following additional filters were then applied to each caller call set:

1) DP > 10
2) QUAL >20
3) AF in normal sample <0.005 or AF in normal sample is at least three times less than AF in tumor sample.

At least two callers should have called a mutation as a somatic to include it into the final somatic call set. The resulting lists of somatic variants were filtered according to the target regions of AmpliSeq Exome Kit provided by the manufacturer and off-target variants were excluded. The final sets for each individual were combined into a single multi-sample VCF file (See Supplementary File 1). Variant annotation, including SIFT and PolyPhen functional effect predictions, was performed with VEP software [12] using data from ENSEMBL release 91.
